# Modeling ovarian hormone effects on glucose–insulin control in type 1 diabetes

**DOI:** 10.1002/btm2.70168

**Published:** 2026-09-15

**Authors:** Nerea Albaladejo‐Carrasco, Clara Furió‐Novejarque, Lía Nattero‐Chávez, Jorge Bondia, José‐Luis Díez

**Affiliations:** ^1^ Instituto Universitario de Automática e Informática Industrial Universitat Politècnica de València Valencia Spain; ^2^ Hospital Universitario Ramón y Cajal Madrid Spain; ^3^ CIBERDEM, ISCIII Madrid Spain

**Keywords:** automated insulin delivery systems, menstrual cycle, ovarian hormones, physiological control, sex‐specific differences, type 1 diabetes

## Abstract

Women with type 1 diabetes mellitus (T1DM) may experience challenges in glycemic control driven by biological sex–specific hormonal fluctuations. Across the menstrual cycle, clinical studies have reported phase‐dependent variations in time in range (TIR), with periods of increased hyperglycemia associated with changes in insulin sensitivity (IS). However, IS is not the sole metabolic process influenced by ovarian hormones; multiple regulatory mechanisms involved in glucose homeostasis appear to be modulated, adding complexity to insulin therapy management in women with T1DM using current automated insulin delivery (AID) systems. In this context, the present work conducts a narrative review of physiologically based mathematical models that seek to couple ovarian hormonal dynamics with glucose–insulin regulation. The analysis reveals a limited number of models that explicitly integrate these interacting systems, highlighting important gaps in the current modeling landscape. These findings underscore the need for physiologically informed models that more accurately represent hormonal–metabolic interactions in women with T1DM, with potential implications for the future development of sex‐specific simulation tools and adaptive closed‐loop insulin delivery strategies.

**Translational Impact Statement**

By identifying key gaps in mathematical models linking menstrual cycle hormones with glucose regulation in women with type 1 diabetes, this work provides a foundation for developing more personalized automated insulin delivery strategies that could improve glycemic control, reduce hyperglycemic episodes, and advance sex‐specific diabetes care.

## 1 INTRODUCTION

Over the past few years, there has been a renewed interest in how biological sex influences human physiology. Men and women exhibit evident anatomical and physiological differences that have been acknowledged across artistic, cultural, and scientific domains. However, the quest to understand not only the visible but also the subtle, systemic differences between sexes has historically been limited. This oversight has led to the underrepresentation of women in multiple scientific and technological fields, including medicine and biomedical engineering, often resulting in conceptual and analytical biases. In clinical research, such biases have contributed to misconceptions regarding female physiology and to the generalization of findings derived predominantly from male or sex‐neutral models.^1^ The consequence has been a significant gap in understanding how biological sex modulates fundamental metabolic and endocrine processes, an omission that has constrained both the efficacy and the equity of therapeutic strategies, particularly in chronic autoimmune diseases.

Type 1 diabetes mellitus (T1DM) represents a paradigmatic immunometabolic disorder in which chronic autoimmune insulin deficiency disrupts systemic physiological homeostasis. The condition impairs the organism's capacity to adapt to dynamic changes in glucose availability, lipid handling, and substrate oxidation. These metabolic perturbations are not isolated phenomena but are intricately intertwined with endocrine regulation, particularly sex steroid signaling. Notably, sex hormones exhibit marked inter‐ and intra‐sex differences in circulating levels, temporal patterns, and biological effects, contributing to sexually dimorphic metabolic responses in T1DM.^2^, ^3^

From a physiological standpoint, understanding the mechanisms that link sex hormones to metabolic regulation is essential to elucidate how these interactions may exacerbate the metabolic imbalances inherent to diabetes. This review does not aim to provide definitive answers to all underlying mechanisms, but rather to highlight critical gaps and future challenges in this field. In this context, key questions arise: what hormonal processes underlie the sex‐specific impact on glucose regulation and related metabolic pathways? Can modern diabetes care continue to overlook physiological differences between sexes without compromising precision and equity?

In women with T1DM, even modest hormonal fluctuations can translate into clinically meaningful changes in IS and glycemic control.^4^, ^5^ However, although both sexes have been included in many glucose regulation studies, sex is most often treated as a static demographic variable rather than as a dynamic physiological factor. Consequently, key sex‐specific determinants of glucose regulation, such as fluctuations in ovarian hormones, cycle‐dependent changes in IS and sex‐specific patterns of lipid and hepatic glucose metabolism, are rarely incorporated into experimental designs or mathematical models. This has resulted in a limited mechanistic understanding of how menstrual cycle–related hormonal variability affects glucose regulation in women with T1DM, with potential implications for the optimization of current glucose management technologies.

The growing recognition of these observations underscores the necessity of integrating sex, and specifically the menstrual cycle, menopause, and gestational period, as key variables in physiological research and in the design of glucose control systems, including closed‐loop insulin delivery technologies.^6^ Understanding and modeling the bidirectional interactions between the ovarian and glucoregulatory axes represent a crucial step toward truly personalized diabetes management in women.

The menstrual cycle constitutes a major source of biological variability, exerting systemic effects that extend well beyond reproductive physiology. Throughout its approximately 28‐day duration, cyclic variations in estradiol (E2) and progesterone (P4) modulate thermoregulation,^7^, ^8^ energy expenditure,^2^ substrate utilization,^9^ and insulin sensitivity.^10^, ^11^ These hormonal oscillations are particularly relevant for women with T1DM, whose glucose regulation depends on finely tuned insulin–glucose feedback mechanisms. Evidence from studies in healthy women demonstrates that these endocrine fluctuations influence glucose kinetics, gastric emptying, incretin secretion, and exercise performance in a phase‐dependent manner.^5^, ^12^, ^13^ E2 has been shown to enhance insulin‐mediated glucose uptake and lipid oxidation, while P4 induces relative insulin resistance and increases hepatic glucose output. The cyclic interplay between these hormones thus generates measurable variations in glycemia, substrate metabolism, and overall metabolic efficiency throughout the menstrual cycle.

Building upon physiological evidence, several studies have reported phase‐dependent variations in glycemia and insulin requirements in women with T1DM.^14^, ^15^, ^16^ However, the magnitude, direction, and timing of these changes remain inconsistent across studies, with some reporting increased insulin resistance or higher glucose levels during the luteal phase, others observing minimal or no systematic phase‐related effects, and substantial interindividual variability often exceeding the average effect size. These discrepancies likely reflect methodological heterogeneity, differences in outcome metrics and cycle phase definitions, and the complex interplay between endocrine, metabolic, and behavioral factors, such as physical activity, stress, dietary patterns, and sleep, which are difficult to comprehensively measure and control in real‐world settings.

Furthermore, it must be acknowledged that the hormonal cycle is inherently individual and varies across women. A range of ovarian disorders can influence menstrual function and, consequently, affect systemic physiology. For instance, polyendocrine metabolic ovarian syndrome (PMOS), a prevalent endocrine disorder, can significantly alter metabolic regulation. As reported by Eleanor et al.,^17^ the pooled prevalence of that reaches nearly 25% among women with T1DM, contributing to hyperandrogenism, a vicious cycle of insulin resistance, and increased insulin doses. Beyond genetic predisposition, factors such as body composition, stress, and mood also modulate hormonal and metabolic responses, leading to menstrual irregularities including amenorrhea or cycle length variations, which may ultimately impair fertility and hormonal balance.^18^

In this context, the coexistence of PMOS and hyperandrogenism reflects a distinctive pathophysiological interplay driven primarily by chronic exposure to supraphysiological peripheral insulin levels rather than classical insulin resistance. Exogenous hyperinsulinemia stimulates ovarian theca cell steroidogenesis and suppresses hepatic sex hormone–binding globulin production, increasing circulating androgen levels. Elevated androgens, in turn, impair insulin sensitivity through tissue‐specific mechanisms, even in the context of autoimmune diabetes. In skeletal muscle, hyperandrogenism reduces insulin‐mediated glucose uptake by disrupting post‐receptor signaling pathways, including IRS‐1/PI3K/Akt activation and GLUT4 translocation. In adipose tissue, androgens promote visceral fat accumulation, enhance lipolysis, and increase free fatty acid flux, contributing to ectopic lipid deposition and metabolic inflexibility. Concomitantly, androgen excess alters adipokine and cytokine profiles, reducing adiponectin and increasing proinflammatory signaling, thereby aggravating peripheral insulin resistance. Hepatic insulin action may also be compromised, with increased gluconeogenesis and impaired suppression of endogenous glucose production. Collectively, these mechanisms establish a vicious cycle in T1DM, whereby exogenous insulin excess drives ovarian hyperandrogenism, which further deteriorates IS, contributing to reproductive dysfunction and amplifying long‐term cardiometabolic and cardiovascular risk.^19^, ^20^, ^21^

Besides its physiological implications, the integration of ovarian hormone dynamics into diabetes research is particularly relevant from a modeling and technological standpoint. Physiological mathematical models are computational representations of biological systems that use mathematical equations, typically differential equations, to describe the mechanisms, feedback loops, and dynamic interactions that govern human physiology. Rather than relying solely on empirical data, these models are grounded in known biological principles, such as hormone secretion patterns, glucose–insulin kinetics, metabolic fluxes, or cardiovascular responses, and play a central role in the development and validation of modern diabetes technologies. This perspective aligns with classical frameworks in physiological systems modeling, as described by Carson and Cobelli,^22^ where mathematical models serve as mechanistic representations of biological function and as the basis for in silico experimentation. These models underpin in silico simulators used to test insulin dosing algorithms, closed‐loop control strategies, and decision‐support systems before their deployment in clinical trials. However, the vast majority of these frameworks, including the widely adopted UVA/Padova simulator,^23^ rely on sex‐neutral assumptions and do not incorporate the cyclical effects of E2 and P4 on insulin sensitivity, glucose uptake, or hepatic glucose production.

In the following sections, we present a comprehensive review of the literature and analytical methodologies aimed at identifying physiological and mathematical models that couple ovarian hormonal dynamics with glucose–insulin regulation, thereby elucidating the mechanistic pathways through which sex hormones modulate metabolic homeostasis in diabetes.

## 2 METHODS

This narrative review synthesizes evidence from peer‐reviewed studies addressing the interplay between menstrual cycle dynamics, glucose metabolism, and insulin sensitivity in both healthy women and those with T1DM. The primary objective was to identify, classify, and critically evaluate physiological, clinical, technological, and mathematical modeling studies exploring the mechanistic links between ovarian hormonal regulation and glucose–insulin homeostasis.

The literature search was conducted using different international databases such as PubMed, Scopus, and Google Scholar. No predefined temporal restriction was applied to the literature search, as this review was conceived as a narrative synthesis rather than a systematic review, with the aim of integrating both foundational and recent evidence relevant to the physiological and technological aspects under study.

A two‐stage search strategy was applied following the criteria described below.

1. Direct, hypothesis‐driven search

The initial phase involved a targeted search using terms directly aligned with the aims of the review, focusing on studies explicitly examining menstrual cycle phases, glucose regulation, and IS. Boolean operators and database‐specific search strings were used to maximize retrieval. The main search terms included were

- “menstrual cycle AND glucose metabolism,”
- “insulin sensitivity AND menstrual phase,”
- “type 1 diabetes AND hormonal variability,”
- “modeling AND ovarian hormones,”
- “estrogens AND progesterone AND glycemic effects AND diabetes,”
- “diabetes AND model AND menstrual cycle,”
- “menstrual cycle AND mathematical model,”
- “sexual hormones AND glucose homeostasis.”

This search captured the core body of research directly addressing the physiological and metabolic consequences of hormonal fluctuations, mainly prioritizing the diabetes context.

The modeling‐specific terms used in the first phase were intentionally broad and partially overlapping across queries, as terminology in this field is highly heterogeneous, such as the description of variations in how menstrual cycle phases, hormonal fluctuations, and metabolic modeling occur. As a result, the same studies were frequently retrieved across multiple search strings.

All records identified in this phase were subsequently deduplicated and screened based on title and abstract to assess relevance to the review's primary objective, namely, studies explicitly addressing menstrual cycle‐related hormonal variability in glucose metabolism and/or IS, particularly within diabetes and modeling contexts. Therefore, the first phase functioned as a comprehensive, hypothesis‐driven retrieval step rather than a strictly partitioned set of distinct sub‐searches, with relevance filtering applied uniformly after aggregation of all retrieved records.

2 Expanded conceptual search

Given the limited availability of studies directly coupling menstrual cycle physiology with T1DM‐specific glucose regulation and mathematical modeling, a second broader search was undertaken. This phase targeted conceptually related domains that could inform or extend the primary research question, including

- hormonal influences on metabolism in women without diabetes,
- sexual dimorphism in glucose homeostasis,
- mathematical or physiological models involving metabolic regulation in diabetes,
- physiological models integrating multi‐hormonal feedback loops related to glycemic homeostasis.

This expanded search was essential for two reasons:

- It identified adjacent research areas addressing partial aspects of the problem, such as metabolism, ovarian physiology, or homeostatic modeling.
- It ensured the inclusion of conceptually relevant contributions necessary to construct a coherent cross‐disciplinary synthesis.

Manual backward and forward citation tracking was conducted for all key papers to ensure completeness. Duplicates and non‐relevant entries were systematically excluded.

Articles were selected based on scientific quality and thematic alignment. Inclusion criteria were as follows:

- Explicit analysis of menstrual cycle phases and their physiological or metabolic consequences.
- Empirical, modeling, or technological studies addressing glucose regulation or insulin sensitivity.
- Research on sexual dimorphism relevant to glycemia or metabolism.
- Quantitative, computational, or simulation‐based evidence linking hormonal dynamics to metabolic outcomes.

Only human‐based research was included to preserve translational relevance. Although a central focus of this review is T1DM, many foundational insights were necessarily extrapolated from non‐diabetic populations due to the scarcity of T1DM‐specific evidence.

Additionally, citation counts and journal impact metrics were used as secondary indicators of scientific influence, ensuring prioritization of rigorously validated and widely recognized studies.

After removing duplicates and excluding publications that did not meet the inclusion criteria, a final corpus of 25 unique studies was obtained. However, given the broad heterogeneity of research, a more granular classification was necessary for the synthesis presented in section 3.

## 3 RESULTS

Following the methodological criteria described above, the selected studies were subsequently grouped by a sub‐thematic classification, which was developed to organize the studies mainly according to their modeling approach, validation type, and clinical or experimental context.

As a result, five heterogeneous tables were constructed to summarize the selected works, differentiating studies by the typology of the model, its validation level, the population and data sources used, the physiological or hormonal effects considered, and the general objective of each model or, in some cases, statistical analysis.

Several studies, listed in Table 1, have described physiologically based models of metabolic regulation that account for sex differences. For example, Abo and Layton proposed a sex‐specific physiological model that captures metabolic differences between men and women during both fasting and feeding states, validated using human metabolic data.^25^ In contrast, Palumbo et al. developed a whole‐body computational model simulating the effects of fuel homeostasis in which sex and age were used to scale VO_2_max and other parameters, rather than explicitly modeling sex‐specific metabolic mechanisms.^26^

**TABLE 1:** Physiological models accounting for sex‐specific differences.

Reference	Modeling approach	Physiological basis (mechanistic)	Apply T1DM	Differentiation by sex	Validation	Cohort/data used	Effects included	General objectives
Abo et al.^24^	Physiological model of energy metabolism	Yes	No	Yes	Experimental data (proof of concept)	Healthy humans (male and female)	CHO and lipid utilization; energy metabolism during exercise	Analyze sex differences in energy metabolism during exercise using a physiological model
Abo et al.^25^	Sex‐specific physiological model of feeding and fasting	Yes	No	Yes	Experimental data	Human data (male and female)	Metabolism of glucose, lipids, and proteins	Analyze metabolic differences by sex in fasting and feeding
Palumbo et al.^26^	Whole‐body model of fuel homeostasis	Yes	No	Yes	Experimental data	Literature data	Metabolites, hormones, substrate use (CHO and fats), exercise intensity effects	Personalize the effect of exercise on metabolism based on individual characteristics

Abbreviation: CHO, carbohydrates.

Within the category shown in Table 2, the models focus primarily on empirical or machine learning (ML) approaches applied to healthy populations. Kilungeja et al. introduced a ML‐based framework capable of identifying menstrual phases using multimodal wearable data such as heart rate, temperature, and sleep metrics, achieving high accuracy in phase detection.^27^ On the other hand, Carvalho et al. conducted a statistical study on active women assessing menstrual phase effects on symptoms and physical performance through validated questionnaires.^28^

**TABLE 2:** Non‐physiological models integrating healthy cohorts and menstrual cycle dynamics.

Reference	Modeling approach	Physiological basis (mechanistic)	Apply T1DM	Differentiation by sex	Validation	Cohort/data used	Effects included	General objectives
Kilungeja et al.^27^	ML model to identify menstrual phases	No	No	Yes	Validated with wearable data	Biometric data	Physiological signals	Detect menstrual phase automatically
de Carvalho et al.^28^	Empirical analysis of symptoms and physical performance	No	No	Yes	Validated with questionnaires	Active women	Menstrual phase, symptoms, physical performance	Assess menstrual cycle impact on physical performance

Abbreviation: ML, machine learning.

In T1DM‐related modeling, several data‐driven studies focused on improving glucose prediction and insulin absorption modeling, as indicated in Table 3. Muñoz‐Organero et al.^29^ and Martínez‐Delgado et al.^30^ both developed ML and deep learning frameworks to learn insulin and carbohydrate absorption patterns from continuous glucose monitoring (CGM) data in T1DM patients, demonstrating enhanced glucose forecasting accuracy. Beyond predictive modeling frameworks, large‐scale retrospective analyses of real‐world AID data have provided valuable insights into glycemic variability under free‐living conditions. For example, Shahid and Lewis analyzed open‐source automated insulin delivery (AID) datasets to characterize interindividual patterns of glycemic outcomes at scale, including stratified analyses by sex.^31^ Although such studies do not propose explicit mathematical or physiological models, they reveal substantial heterogeneity in glycemic control achieved with current AID systems, highlighting limitations that may not be captured by model‐based approaches alone.

**TABLE 3:** Data‐driven approaches and large‐scale analyses applied to T1DM.

Reference	Modeling approach	Physiological basis (mechanistic)	Apply T1DM	Differentiation by sex	Validation	Cohort/data used	Effects included	General objectives
Muñoz‐Organero et al.^29^	DL Model for absorption and digestion	No	Yes	No	Validated with CGM data	T1DM patients	Insulin and CHO absorption	Learn digestion and absorption dynamics
Martínez‐Delgado et al.^30^	ML model including insulin and CHO absorption	No	Yes	No	Validated with CGM data	T1DM patients	Insulin and carbohydrate absorption, glucose prediction	Improve glycemic prediction
Shahid et al.^31^	Large‐scale analysis of AID data	No	Yes	Yes	Validated with big data	Open‐source data	Glycemic variability and control patterns	Evaluate automated insulin delivery performance at scale

Abbreviations: AID, automated insulin delivery; CGM, continuous glucose monitoring; CHO, carbohydrates; DL, deep learning; ML, machine learning.

The next subgroup, illustrated in Table 4, encompassed studies explicitly modeling female reproductive or hormonal processes, some extending toward metabolic implications.

**TABLE 4:** Physiological models capturing sex‐specific differences and menstrual cycle effects.

Reference	Modeling approach	Physiological basis (mechanistic)	Apply T1DM	Differentiation by sex	Validation	Cohort/data used	Effects included	General objectives
Fischer et al.^32^	Mathematical model of the ovarian cycle	Yes	No	Yes	Conceptual	Women undergoing IVF treatment	E2, LH, FSH, P4	Optimize ovarian stimulation protocols
Ramírez^33^	Semi‐physical metabolic–ovarian model	Yes	No	Yes	Conceptual and experimental	Healthy women	IS, insulin secretion, β‐cell mass	Study E2 effects on glucose homeostasis
Graham et al.^34^	Reduced model of the female endocrine axis	Yes	No	Yes	Validated with hormonal data	Healthy women	E2, P4, LH, FSH	Describe female endocrine variability
Manrique‐Córdoba et al.^35^	Glucose–insulin model including menstrual cycle	Yes	No	Yes	Experimental data	Healthy women	Sex hormones, IS	Model hormonal influence on glycemic regulation
Shack et al.^36^	HPO axis model	Yes	No	Yes	Conceptual	Literature parameters	GnRH, LH, FSH, E2, P4	Reproduce menstrual hormonal oscillations
Graham et al.^37^	Endocrine dynamic feedback model	Yes	No	Yes	Conceptual	Literature parameters	Hyperinsulinemia, testosterone	Explore mechanisms of hyper‐androgenism and ovulatory dysfunction
Vallée et al.^38^	Semi‐mechanistic multi‐hormonal HPO model	Yes	No	Yes	Conceptual	Virtual cohort (550 women)	Multi‐hormonal phenotypes.	Generate realistic, synthetic, hormonal time series
Hendrix et al.^39^	Nonlinear physiological menstrual cycle model	Yes	No	Yes	Experimental data (proof of concept)	Literature parameters	LH, FSH, E2, P4, ovulatory patterns	Assess androgen influence on cycle dynamics
Kim et al.^40^	Biochemical metabolic model	Yes	No	Yes	Experimental data (proof of concept)	Literature data	Metabolism of one‐carbon, folate, methionine, hormonal variations during the menstrual cycle and pregnancy	Simulate metabolic effects of menstrual cycle and pregnancy

Abbreviations: FSH, follicle‐stimulating hormone; GnRH, gonadotropin‐releasing hormone; HPO, hypothalamic–pituitary–ovarian; IS, insulin sensitivity; IVF, in vitro fertilization; LH, luteinizing hormone.

Fischer and Röblitz developed a mechanistic model^32^ of the ovarian cycle describing E2, luteinizing hormone (LH), follicle‐stimulating hormone (FSH), and P4 dynamics, validated in women undergoing in vitro fertilization (IVF) treatment. Carolina Ramíırez et al. proposed a semi‐physical model coupling metabolic and ovarian subsystems to explore how E2 modulates glucose homeostasis, insulin secretion, and β‐cell mass in healthy women.^33^ Graham et al. designed a reduced mathematical representation of the female endocrine axis, capturing cyclic variability of key reproductive hormones.^34^

Particularly relevant to glucose metabolism, Manrique‐Córdoba et al.^35^ formulated a glucose–insulin physiological model modulated by menstrual phase, providing a conceptual framework for quantifying hormonal influences on IS in women without diabetes.

Additionally, earlier endocrine models such as Shack et al. described the hypothalamic–pituitary–ovarian (HPO) axis dynamics, laying the conceptual groundwork for later integrations of metabolic and reproductive control systems.^36^

Table 5 includes foundational physiological models that have shaped diabetes modeling and simulation research. In particular, the Bergman Minimal Model (1981)^45^ and the Sorensen model (1985)^41^ established the theoretical groundwork for later frameworks, most notably the models proposed by Hovorka et al.^42^ and Dalla Man et al.,^43^ which could then be effectively applied to the treatment, management, and in silico simulation of diabetes. Later extensions, such as Kovatchev^44^ and Visentin et al.,^48^ validated these models in silico and clinically to support closed‐loop control algorithms. Others, as Mosekilde provided an early kinetic model of subcutaneous insulin absorption, addressing new physiological effects in glucose‐insulin dynamics.^47^

**TABLE 5:** Physiological and metabolic models in diabetic and non‐diabetic populations.

Reference	Modeling approach	Physiological basis (mechanistic)	Apply T1DM	Differentiation by sex	Validation	Cohort/data used	Effects included	General objectives
Sorensen^41^	Comprehensive physiological model	Yes	Yes	No	Clinical literature	Literature data	Multiple metabolic compartments	Improve insulin therapy design
Hovorka et al.^42^	Nonlinear predictive control model	Yes	Yes	No	Clinically validated	T1DM subjects	Glucose, insulin, meals	Glucose control using MPC
Dalla Man et al.^43^	Extended meal–glucose–insulin model	Yes	Yes	No	Conceptual and experimental	UVa/Padova simulator	Meals, insulin kinetics, physical activity	Assess the impact of physical activity on postprandial glucose and evaluate insulin dosing strategies in T1DM
Kovatchev et al.^44^	Multicompartment physiological model	Yes	Yes	No	Experimental data (proof of concept)	UVa/Padova simulator	Glucose, insulin, meals	Validate closed‐loop systems
Bergman et al.^45^	Minimal glucose–insulin model	Yes	No	No	Clinically validated	Human subjects	Glucose–insulin dynamics	Quantify IS
Díaz et al.^46^	In silico IS model across menstrual cycle	Yes	Yes	Yes	In silico validation using clinical data	Simulated women	Insulin sensitivity, TIR impact	Assess cycle‐dependent technological adjustments per cycle
Mosekilde et al.^47^	Kinetic model of subcutaneous insulin absorption	Yes	Yes	No	Experimental data	Human subjects	Subcutaneous insulin absorption	Model insulin PK
Visentin et al.^48^	UVa/Padova simulator extension (24 h)	Yes	Yes	No	In silico validation against clinical data	UVa/Padova simulator	Meals, exercise, sleep	Simulate full day glucose control in T1DM

Abbreviations: IS, insulin sensitivity; MPC, model predictive control; PK, pharmacokinetics.

Among more recent advances, Díaz et al.^46^ proposed an in silico model of insulin replacement in women with T1DM that incorporates cycle‐dependent variations in IS, derived from euglycemic clamp data. This approach provides one of the few attempts to account quantitatively for menstrual cycle–related changes in glycemic regulation, although it does not explicitly model individual ovarian hormones such as E2 or P4.

## 4 DISCUSSION

Overall, this literature review reveals a marked imbalance between the high methodological maturity of existing glucose–insulin models for T1DM and the scarce integration of ovarian hormonal regulation within these frameworks. Despite the extensive validation and clinical adoption of both physiological and data‐driven T1DM models, hormonal variability, particularly that associated with the menstrual cycle, is typically treated as residual variability or as an implicit, unmodeled disturbance rather than as a systematic, biologically driven input influencing glucose homeostasis.

In recent years, several modeling efforts have begun to incorporate sex differences as relevant determinants of metabolic regulation, aiming to generate more physiologically realistic simulations. However, most of these models still fail to account for intra‐individual hormonal fluctuations, and even fewer have been developed specifically in a diabetes‐focused context. Furthermore, models designed to detect or classify menstrual cycle phases generally rely on indirect biometric proxies rather than on direct hormonal measurements and often treat these variables as independent predictors without embedding their mechanistic endocrine pathways.

Classical models, including those developed by Bergman et al.,^43^ Hovorka et al.,^41^ and Dalla Man et al.,^47^ have established robust mathematical representations of glucose–insulin dynamics, yet remain sex‐neutral and insensitive to cyclical fluctuations in E2, P4, or androgens. As a result, they cannot reproduce the phase‐dependent glycemic variability observed in some women with T1DM, including luteal‐phase insulin resistance, increased basal requirements, or altered postprandial responses reported in clinical studies.

This limitation extends to AID technologies. Although clinical studies have evaluated AID performance across different menstrual cycle phases and, in some cases, reported no statistically significant differences in overall glycemic outcomes,^49^ while others have reported contrasting results, particularly with respect to TIR,^50^ current AID algorithms do not incorporate explicit hormonal modeling, nor has phase‐adaptive control been clinically validated as a strategy to improve glycemic control across the menstrual cycle.

Only a small number of recent contributions, most notably those by Manrique‐Córdoba et al.,^35^ Díaz et al.,^46^ and Carolina Ramírez et al.,^33^ have begun to bridge this gap by proposing semi‐physiological and in silico modeling frameworks that explicitly link menstrual‐cycle dynamics to IS and/or glucose uptake, thereby enabling preliminary exploration of phase‐dependent metabolic and therapeutic adaptations. Table 6 synthesizes these approaches in a comparative theoretical overview, highlighting how each framework conceptualizes menstrual‐cycle effects, the metabolic processes they target, and the level of abstraction at which cycle‐related modulation is introduced.

**TABLE 6:** Comparative theoretical overview of glucose–insulin models incorporating menstrual cycle–related variability.

Model/reference	Baseline glucose–insulin framework	Cycle representation	Targeted metabolic process	Modified term (explicit)	Mathematical form of modulation
Manrique‐Córdoba et al.^35^	Oral glucose minimal model (Dalla Man et al.^51^)	Implicit, via cycle day (cDay); no explicit E2/P4 dynamics	Peripheral IS (glucose uptake)	P2U	$P2U\left(\text{cDay}\right)=P2U_{0}+P2U_{0}\left(-\tanh\left(\frac{\text{cDay}-14}{2}\right)\right)$
			Insulin‐dependent glucose utilization	*U*_id_(*t*) via dependence on *X*(*t*)	$U_{\mathrm{id}}\left(t\right)=\frac{V_{m}\left(X\left(t\right)\right)\;G_{t}\left(t\right)}{K_{m}\left(X\left(t\right)\right)+G_{t}\left(t\right)}$
Díaz et al.^46^	UVA/Padova T1D simulator	Discrete, two‐phase (follicular vs. luteal)	Global IS as a subject‐specific trait	$VR_{\text{IS}}$ (IS variability ratio) $\mathrm{Ph}1\;\left(\text{Baseline Phase}\right)$ $\mathrm{Ph}2\;\left(\text{Other Phase}\right)$	$VR_{\text{IS}}=\frac{\text{AUGI}R_{\mathrm{Ph}2}}{\text{AUGI}R_{\mathrm{Ph}1}}$
			Insulin therapy parameters (the dosing parameters modified to account for cycle related IS changes)	BR (basal rate) CR (carbohydrate‐to‐insulin ratio) CF (correction factor)	$BR_{\mathrm{Ph}2}=\frac{1}{a}BR_{\mathrm{Ph}1}$ $CR_{\mathrm{Ph}2}=a\;CR_{\mathrm{Ph}1}$ $CF_{\mathrm{Ph}2}=a\;CF_{\mathrm{Ph}1}$
Ramírez^33^	Minimal glucose–insulin–β‐cell model (Topp et al.^52^) coupled to menstrual cycle model	Explicit, via dynamic E2 and P4	IS	$\mu_{\max,\mathrm{up}}\left(E_{2},P_{4}\right)$	$\mu_{\max,\mathrm{up}}\left(E_{2},P_{4}\right)=k_{I\mathrm{up}}\frac{E_{2}}{K_{sE_{2}}+E_{2}}\frac{K_{sP_{4}}}{K_{sP_{4}}+P_{4}}$
			Insulin secretion	${\mathrm{Ins}}_{\sec}\left(E_{2},P_{4},G\right)$	Monod‐type saturation functions
			β‐cell mass dynamics	$\mu_{\beta ,\text{growth}}\left(E_{2},G\;\right)-\mu_{\beta ,\text{apop}}\left(G\right)$	Hormone‐dependent growth/apoptosis terms

*Note*: P2U(cDay) is proportional to insulin‐dependent glucose utilization *U*_id_(*t*) through the insulin action state *X*(*t*), i.e., *X*(*t*) ∝ P2U(cDay).

Abbreviation: P2U, rate of insulin action on peripheral glucose utilization.

A first group of approaches operates at the level of parametric modulation of physiological glucose–insulin models. In this category, Manrique‐Córdoba et al.^35^ introduce cycle‐dependent variability by modifying a single key parameter governing peripheral insulin action within the oral glucose minimal model. The menstrual cycle is represented implicitly through cycle day, without modeling E2 or progesterone P4 dynamics. Conceptually, the cycle is treated as a smooth, continuous source of variability that modulates insulin effectiveness, while the underlying metabolic structure of the model remains unchanged. This strategy preserves the interpretability and simplicity of the classical Dalla Man^52^ framework, allowing cyclical fluctuations in glucose uptake to be explored without increasing model dimensionality. However, the absence of explicit hormonal states limits its ability to distinguish mechanistic contributions of individual ovarian hormones.

A second class of models, exemplified by Díaz et al.,^46^ adopts a control‐oriented abstraction, in which menstrual cycle effects are not embedded within the internal physiology of the glucose–insulin system but are instead translated into phase‐dependent adaptations of insulin therapy parameters. Here, the cycle is discretized into two phases, and IS variability is treated as a subject‐specific trait derived from clamp data. Rather than modifying glucose–insulin dynamics directly, the framework adjusts basal rate, carbohydrate ratio, and correction factor to compensate for inter‐phase changes in IS. This approach is particularly relevant for AID systems, as it directly informs control parameters. Nevertheless, it remains agnostic to the biological mechanisms underlying cycle‐related IS changes and does not track hormonal dynamics explicitly.

In contrast, Carolina Ramírez^33^ represents a mechanistically integrated, hormone‐driven modeling strategy, coupling a phenomenological ovulatory–menstrual cycle model with a minimal glucose–insulin–β‐cell system. In this framework, E2 and P4 are dynamic state variables that modulate multiple metabolic processes, including IS, insulin secretion, and β‐cell mass turnover, the latter being characteristic of non‐diabetic phenotypes. Cycle effects are encoded through saturating nonlinear functions that capture synergistic and antagonistic hormone interactions. Conceptually, this model operates at a lower level of abstraction, embedding ovarian hormone dynamics directly into the structure of glucose regulation. While this enables a richer and more physiologically grounded representation of hormone–metabolism interactions, the model remains largely exploratory and has not yet been clinically validated in women with T1DM.

Additionally, these three approaches also differ substantially in their degree of validation and translational maturity. Díaz et al.^46^ constitutes the most clinically grounded framework, as it integrates clamp‐derived physiological evidence into a validated diabetes simulator to assess the impact on insulin delivery. Nevertheless, its conclusions are confined to in silico evaluation and require clinical confirmation for real‐world translation. On the other hand, Manrique‐Córdoba et al.^35^ occupies an intermediate position, supported by experimental validation but still limited in mechanistic and clinical generalization. In contrast, Ramírez^33^ remains an exploratory, mechanistic model that, while physiologically rich, requires further validation before its potential clinical relevance in T1DM can be established. This gradient of validation highlights a continuum from control‐oriented applications to mechanistic hypothesis‐generating frameworks.

Taken together, the models summarized in Table 6 span a spectrum of modeling abstraction, ranging from implicit, continuous modulation of physiological parameters through discrete, therapy‐level control adaptations to explicit, peripheral hormone‐driven mechanistic integration. While each of these approaches offers complementary insights, none simultaneously provides hormonal explicitness, clinical validation in T1DM populations, and direct applicability to closed‐loop insulin delivery. Consequently, despite recent conceptual advances, the literature still lacks clinically validated, control‐oriented frameworks capable of identifying, tracking, and integrating ovarian hormonal dynamics into insulin delivery algorithms in women with T1DM from a physiological perspective.

## 5 CONCLUSION

Although the field is rapidly evolving, the studies discussed here represent only a fraction of the broader research addressing elements relevant to hormone‐aware metabolic models. Importantly, this review also emphasizes the numerous aspects of sexual dimorphism that remain unaccounted for in current modeling efforts. While focusing on the mathematical integration of two physiological systems, glucose regulation and ovarian endocrinology, traditionally treated as independent, other biological pathways likely interact with both systems and may meaningfully influence the resulting metabolic phenotype.

Clinical evidence also demonstrates substantial inter‐ and intra‐individual variability in glycemic responses across menstrual phases, indicating that hormonal fluctuations alone cannot fully explain the observed patterns. Lifestyle factors, behavioral routines, psychosocial context, ethnicity, geographical environment, and emotional state may all contribute to this variability and warrant further investigation.

Moreover, the inconsistent findings across clinical studies, including within the same individual across cycles, highlight major gaps that must be addressed, particularly within the T1DM population, where therapeutic decisions have immediate and profound effects on glucose regulation. The lack of mechanistic models that capture these multidimensional interactions represents a significant barrier to advancing personalized insulin therapy.

In summary, considering the aspects discussed above, more personalized therapeutic approaches are needed, incorporating individual‐specific factors and further exploring clinically relevant clusters of sex‐related physiological variation, particularly in women, to improve treatment experiences and clinical outcomes. Progress in this direction will depend on the development of clinically validated, control‐oriented frameworks capable of identifying, tracking, and integrating ovarian hormonal dynamics into insulin delivery algorithms by incorporating endocrine pathways that more accurately reflect the underlying physiology.

## AUTHOR CONTRIBUTIONS

**Jorge Bondia:** Project administration; supervision; funding acquisition. **Lía Nattero‐Chávez:** Supervision; resources. **Clara Furió‐Novejarque:** Conceptualization; writing – review and editing; supervision. **Nerea Albaladejo‐Carrasco:** Conceptualization; methodology; investigation; writing – original draft; writing – review and editing. **José‐Luis Díez:** Conceptualization; supervision; visualization; funding acquisition.

## FUNDING INFORMATION

Grant PID2022‐137723OB‐C21 funded by MICIU/AEI/10.13039/501100011033 and by “ERDF/EU.” The funder did not have any role in study design, data collection and analysis, decision to publish, or preparation of the manuscript.

## CONFLICT OF INTEREST STATEMENT

The authors declare no conflicts of interest.

## Data Availability

Data sharing not applicable to this article as no datasets were generated or analysed during the current study.
